# The viability of ABO-incompatible kidney transplants: a single-center cohort in China

**DOI:** 10.3389/fimmu.2026.1747411

**Published:** 2026-02-17

**Authors:** Yiding Chen, Jiashan Pan, Dongsheng Li, Kun Wang, Handong Ding, Fei Zhang, Wenbo Wang, Pengfei Li, Jinbiao Zhong, Guiyi Liao

**Affiliations:** 1 Department of Urology, The First Affiliated Hospital of Anhui Medical University, Hefei, China; 2 Institute of Urology, Anhui Medical University, Hefei, China; 3 Anhui Province Key Laboratory of Urological and Andrological Diseases Research and Medical Transformation, Anhui Medical University, Hefei, China

**Keywords:** ABO-incompatible living donor kidney transplantation, desensitization, graft survival, meta-analysis, postoperative outcomes

## Abstract

**Background:**

ABO-incompatible living donor kidney transplantation (ABOi-LDKT) offers a potential solution to organ shortages, but its safety and economic impact in China remain uncertain. This study evaluated short- to mid-term outcomes of ABOi-LDKT in a Chinese population by integrating evidence from a meta-analysis and a single-center cohort.

**Methods:**

We performed a meta-analysis of 18 studies including 15,611 kidney transplant recipients to compare graft and patient survival between ABOi-LDKT and ABO-compatible LDKT (ABOc-LDKT). In parallel, we conducted a retrospective single-center cohort study of 41 ABOi-LDKT and 132 ABOc-LDKT recipients transplanted between 2021 and 2022. Outcomes included patient and graft survival, renal function, postoperative complications, infections, delayed graft function (DGF), acute rejection, and hospitalization costs.

**Results:**

The meta-analysis showed lower 1-year graft survival and 3-year patient survival in ABOi-LDKT than in ABOc-LDKT. In contrast, our single-center cohort demonstrated comparable 1- and 3-year patient and graft survival between groups. No significant differences were observed in surgical complications, infections, DGF, or acute rejection. ABOi-LDKT recipients showed better renal function at two weeks post-transplant, with no sustained differences thereafter. Hospitalization costs were higher in the ABOi-LDKT group, reflecting additional desensitization procedures.

**Conclusion:**

In this Chinese single-center cohort, ABOi-LDKT achieved short- to mid-term clinical outcomes comparable to ABOc-LDKT despite higher upfront costs. When combined with contemporary desensitization protocols, ABOi-LDKT appears to be a safe and feasible strategy to expand access to living donor kidney transplantation in settings with severe organ shortages.

## 1 Introduction

Kidney transplantation remains the gold standard for end-stage renal disease (ESRD), yet the scarcity of compatible organs severely restricts its application (1–3). ABO-incompatible living donor kidney transplantation (ABOi-LDKT) has emerged as a critical strategy to expand the donor pool, with reports suggesting it can increase living donation by up to 30% (4). Historically, the “viability” of ABOi-LDKT was challenged by high rates of hyperacute rejection. However, the evolution of desensitization protocols—transitioning from splenectomy to Rituximab-based regimens combined with plasmapheresis (PE)—has revolutionized outcomes, enabling graft survival rates comparable to ABO-compatible living donor kidney transplantation (ABOc-LDKT) (5, 6).

Despite these advances, the broad viability of ABOi-LDKT in developing regions remains debated, centering on three critical pillars: effectiveness (successful antibody depletion and prevention of rebound), safety (balancing desensitization against infection risks), and economy (the cost-benefit ratio of pre-conditioning). In clinical practice, heterogeneous desensitization protocols regarding Rituximab dosing and target isoagglutinin titers have led to varying outcomes, particularly concerning infection-related mortality and antibody-mediated rejection (AMR) (7, 8). Furthermore, the economic burden of desensitization in self-pay or mixed-payer healthcare systems poses a barrier to widespread adoption.

To comprehensively assess the viability of ABOi-LDKT, this study adopts a dual approach. We integrated a systematic meta-analysis of Asian cohorts to establish regional benchmarks for safety and efficacy. Concurrently, we conducted a single-center retrospective study to rigorously validate an individualized desensitization protocol. Specifically, we focused on the effectiveness of CD19^+^ B-cell targeted Rituximab dosing in reducing isoagglutinin titers, the safety of stratified infection prophylaxis in mitigating perioperative complications, and the economic implications of these interventions. This study aims to provide robust evidence that ABOi-LDKT is a viable, safe, and cost-effective therapeutic option for expanding renal transplantation access in China.

## 2 Material and method

### 2.1 Search strategy and selection criteria

A systematic literature search was conducted across international and Chinese databases, including Web of Science, Embase, PubMed, Google Scholar, CNKI, Baidu Scholar, and Wanfang. The search window spanned from January 2001 to April 2024. Keywords and Medical Subject Headings (MeSH) included “kidney transplantation,” “ABOi,” “renal transplantation,” “ABO incompatible,” “ABOi-rTx,” “ABOi-KT,” and “ABOi-LDKT”.

Study eligibility was determined using the PICO framework to ensure relevance and homogeneity:

Population (P): Asian patient populations undergoing living donor kidney transplantation.

Intervention (I): ABOi-LDKT utilizing a Rituximab-based desensitization protocol.

Comparator (C): ABOc-LDKT.

Outcomes (O): Clinical outcomes including patient and graft survival with a minimum follow-up duration of 1 year, providing sufficient data for extraction.

### 2.2 Data extraction and quality assessment

Data were extracted independently by two investigators using a standardized form. Extracted variables included lead author, publication year, follow-up duration, dialysis history, sample sizes, and event counts (death/graft loss) at 1, 3, and 5 years. Quality assessment was performed using the Newcastle-Ottawa Scale (NOS). (http://www.ohri.ca/programs/clinical_epidemiology/oxford.asp). Any discrepancies in data extraction or quality scoring were resolved through discussion or by consulting a third senior investigator.

### 2.3 Meta-analysis statistical methods

Meta-analysis was performed using Stata 12.0 (StataCorp, College Station, TX, USA). Pooled Odds Ratios (ORs) with 95% Confidence Intervals (CIs) were calculated for graft and patient survival. Subgroup analyses evaluated complications including Cytomegalovirus (CMV)/BK virus infection and acute rejection. Heterogeneity was assessed via the Cochrane *Q* test and *I^2^* statistic. To account for potential between-study variance, a random-effects model was applied if significant heterogeneity was observed (P < 0.1 or *I^2^* > 50%); otherwise, a fixed-effects model was utilized. Publication bias was assessed using Begg’s test and funnel plots, and sensitivity analysis was conducted to verify result stability.

### 2.4 Inclusion criteria for the receiver in clinical analysis

Recipients in both groups were required to meet the following criteria: (1) follow-up duration of >1 year in our outpatient department; (2) ≤3 human leukocyte antigen (HLA) mismatches; (3) complement-dependent cytotoxicity (CDC) <10%; (4) negative reactivity for class I and class II antibodies; (5) first kidney transplant from a direct blood relative who had provided written informed consent for altruistic donation; (6) adult recipients; and (7) implantation of the graft in the right iliac fossa. Recipients with left iliac fossa implantation (n = 15) were excluded *a priori* to reduce surgical and technical heterogeneity (e.g., vascular length and angle, ureteral course, and wound exposure), as these factors could influence early non-immunologic complications in our small cohort.

### 2.5 Desensitization protocol (ABOi-LDKT group only)

The experimental group received desensitization therapy based on the Technical Operating Specifications for ABO-Incompatible Living Donor Kidney Transplantation in China (2019) (7). An individualized Rituximab regimen was administered based on preoperative CD19^+^ B-cell counts: patients with >15% received 200 mg at 4 weeks, 100 mg at 2 weeks, and 100 mg at 1 day preoperatively; those with 10–15% received 100 mg at these three time points; and those with <10% received 100 mg at 4 and 2 weeks preoperatively. Isoagglutinin titers (IgM and IgG) were monitored at initial typing, before and after pharmacotherapy, before and after PE, and on postoperative days 2, 5, and 14. The target titer was <1:16. If titers exceeded 1:16, PE or Double Filtration Plasmapheresis (DFPP) was performed using albumin or fresh frozen plasma as replacement fluids until the target was met.

### 2.6 Immunosuppression and perioperative management

While the ABOc-LDKT group did not receive immunosuppressants before surgery, the ABOi-LDKT group initiated triple therapy 1–2 weeks preoperatively, consisting of Tacrolimus (0.08–0.1 mg/kg/day, target serum trough 10–12 ng/ml), Mycophenolate Mofetil (MMF, 1.0–1.5 g/day, target AUC 30–60 ng/ml), and Prednisone (20 mg/day). Induction therapy for all patients included Antithymocyte Globulin (ATG, 25 mg on days 0, 1, and 2) and methylprednisolone sodium succinate (500 mg IV on days 0, 1, and 2). Postoperatively, methylprednisolone was tapered daily to 120 mg, 80 mg, and 40 mg on days 3, 4, and 5, respectively, and switched to oral prednisone (10 mg/day) on day 6. MMF (1.0–1.5 g/day) was resumed on the evening of surgery, and oral Tacrolimus (initial dose 0.1–0.2 mg/kg/day) was started on postoperative day 3, adjusted to maintain therapeutic levels.

### 2.7 Perioperative management: infection prophylaxis

Routine antibacterial prophylaxis utilizing Mezlocillin sodium/Sulbactam sodium was administered from postoperative day (POD) 0 to 12 targeting Gram-negative bacteria; coverage for fungi or Gram-positive bacteria was reserved for culture-positive cases derived from donor perfusion or drainage fluid analysis. Prophylaxis against Pneumocystis jirovecii pneumonia was initiated on POD 7 with Sulfamethoxazole-Trimethoprim (SMZ-TMP) (200/40 mg daily) and continued for 6 months. Ganciclovir was administered for CMV prophylaxis from POD 7 to month 3, with dosages strictly stratified by estimated glomerular filtration rate (eGFR): (1) eGFR >70 mL/min: 1000 mg PO three times daily (tid); (2) eGFR 50–69 mL/min: 500 mg PO tid; (3) eGFR 25–49 mL/min: 500 mg PO twice daily (bid); (4) eGFR 10–24 mL/min: 500 mg PO once daily (qd); (5) eGFR <10 mL/min: 500 mg PO every other day (qod).

### 2.8 Clinical data collection and outcome definitions

Comprehensive clinical data were systematically collected for recipients, including demographics, preoperative history, dialysis modality, and perioperative metrics such as hospital stay and costs. Specific surveillance outcomes included the incidence of severe pulmonary and urinary tract infection (UTI) requiring re-hospitalization within 1 year post-transplantation, while acute rejection episodes and surgery-related complications were monitored during the perioperative period. Follow-up assessments tracked patient and graft survival at 7 days, 14 days, 1 month, 6 months, and 1 year post-transplantation. Key outcomes were defined as follows: delayed graft function (DGF) was defined as the requirement for dialysis within the first week post-transplantation; Acute Rejection was suspected based on clinical indicators and confirmed by percutaneous renal biopsy according to the Banff classification; Surgical complications were categorized as ureteral stenosis, anastomotic hemorrhage, or wound infection/dehiscence; and postoperative renal function was assessed using the CKD-EPI creatinine equation (race-free) reported as eGFR(mL/min/1.73 m²).

Donor demographics and baseline characteristics were recorded following the recipient protocol. Preoperative donor glomerular filtration rate (GFR) was measured via renal dynamic scintigraphy using the Gates camera method with ^99m^*Tc-DTPA.* Net injected counts were obtained by syringe pre/post counting, and regions of interest (ROIs) were drawn over each kidney with background subtraction. Attenuation was corrected using Tonnesen depths (YL = 13.2×W/H+0.7; YR = 13.3×W/H+0.7; μ = 0.153 cm^−1^), and the 2–3-min window provided bilateral uptake (%). Total GFR was calculated as GFR_total_ (mL/min) = 9.813×uptake (%) − 6.825, with single-kidney GFR = GFR_total_ × split function; values were BSA-standardized to 1.73 m².

### 2.9 Statistical analysis

Data analyses were performed using R v4.2.2 (R Foundation, Vienna, Austria) and GraphPad Prism v9.0 (GraphPad Software, San Diego, CA, USA). Continuous variables were expressed as mean ± SD or median (IQR) and tested for normality using the Kolmogorov–Smirnov test (P < 0.05 indicated non-normal distribution). For group comparisons, normally distributed continuous variables were analyzed using the unpaired Student’s t-test, while non-normally distributed variables were assessed using the Mann–Whitney U test. To evaluate longitudinal within-group changes in serum creatinine (Scr) and eGFR at different follow-up time points, paired t-tests were employed. Categorical data were compared using the Chi-square test or Fisher’s exact test as appropriate. Survival models were constructed using the “survival” and “survminer” R packages to generate Kaplan–Meier curves, with differences assessed via the log-rank test. While propensity score methods were considered to balance baseline covariates, the modest sample size of the ABOi-LDKT cohort after prespecified exclusions would have led to unstable estimates. Therefore, we focused on strict eligibility criteria and standardized perioperative management to minimize major confounding in this exploratory cohort. A two-tailed P-value < 0.05 was considered statistically significant.

## 3 Results

### 3.1 Meta-analysis results

**Figure 1:**
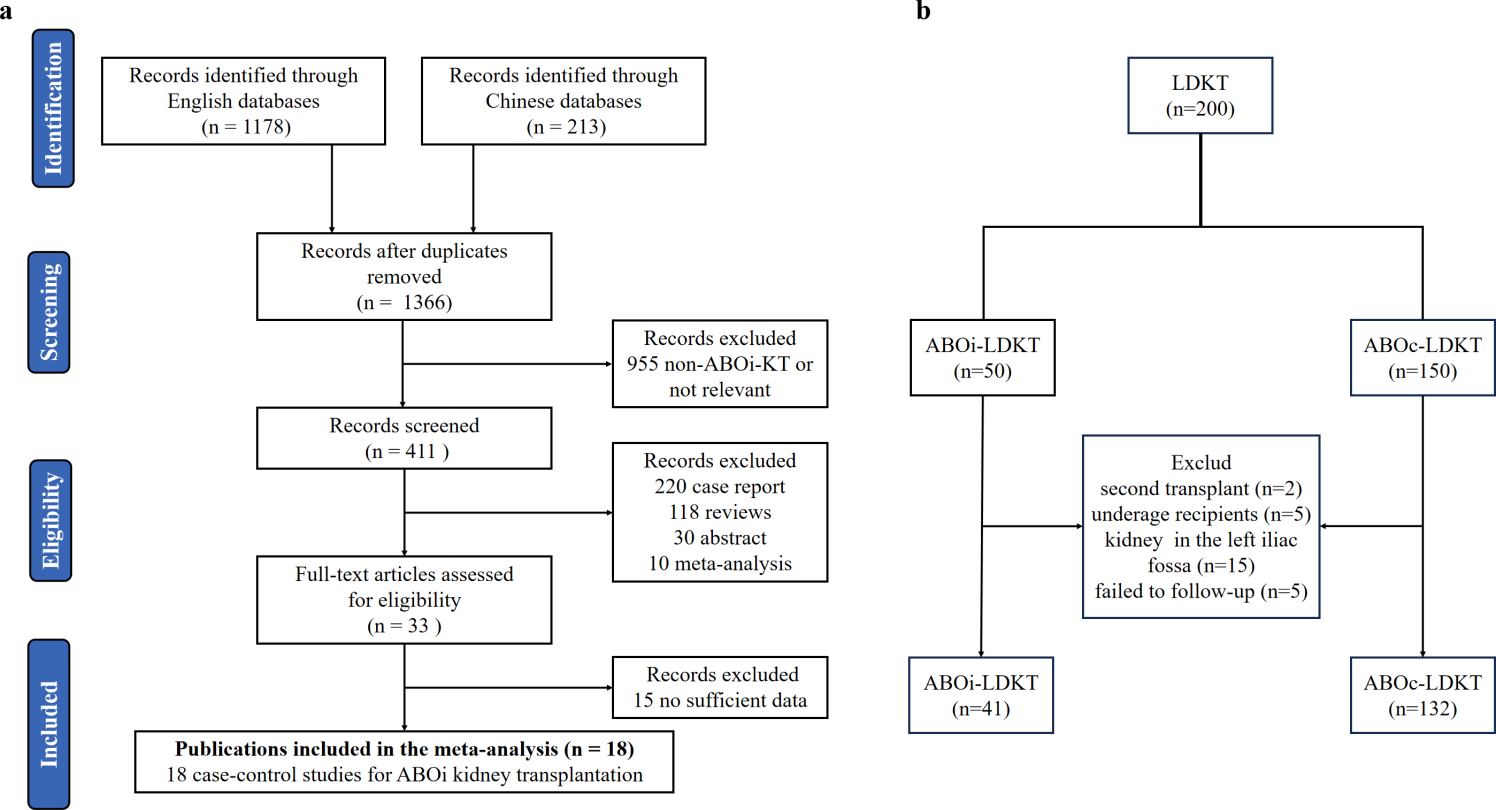
Flowchart for the current study. **(a)** Process of the literate selection of the meta-analysis. **(b)** Process of the collection of clinical samples.

The selection process used in this study is illustrated in **Figure 1A**. A total of 18 papers were included in this meta-analysis, consisting of 17 in English (6, 9–24) and 1 in Chinese (8). All studies were published between January 1, 2001, and April 1, 2024, involving 15,611 patients (**Figure 1a**). **Table 1** summarizes the detailed information on the studies included in this meta-analysis. All included papers were scored 5 or higher on the NOS scale.

**Table 1:** Summary of included cohorts: ABO-compatible and ABO-incompatible.

Author	Country	Year	ABOc-LDKT					ABOi-LDKT					Research type
			n	Follow-up time	Mean age	BMI	Dialysis time	n	Follow-up time	Mean age	BMI	Dialysis time	
Tanabe et al. (6)	Japan	2009	55	NA	38.9 ± 12.5	20.8 ± 2.6	43 (16-86)	24	NA	43.0 ± 13.5	21.4 ± 2.4	27 (19-66)	RCT
Jeon et al. (9)	South Korea	2010	61	NA	NA	NA	NA	22	NA	45 (33-61)	NA	NA	RCT
Fuchinoue et al. (10)	Japan	2011	280	36.4 ± 19.57	43.5 ± 13.7	NA	39.7 (0–304.8)	50	36.4 ± 19.6	52.6 ± 12.1	NA	31.2 (0.7–184.6)	RCT
Kohei et al. (11)	Japan	2012	83	NA	40.4 ± 12.6	NA	44 (16–86)	57	NA	44.0 ± 14.8	NA	26 (19–57)	RCT
Hwang et al. (12)	South Korea	2013	138	31	42.8 ± 11.2	22.9 ± 3.6	19.8 ± 32.1 (n=103)	35	31	44.1 ± 9.2	22.9 ± 3.6	27.2 ± 35.3 (n=25)	RCT
Takahashi et al. (13)	Japan	2013	6716	NA	NA	NA	NA	1427	NA	NA	NA	NA	RCT
Ashimine et al. (14)	Japan	2014	228	36	40.0 ± 15.1	NA	NA	30	36	49.0 ± 15.1	NA	NA	RCT
Shin et al. (15)	South Korea	2015	396	46 (1-65)	43 (7-73)	22.2 (12.3-39.7)	7 (0-207)	73	39(2-63)	44 (20-64)	22.8 (16.1-31.0)	10 (0-252)	RCT
Okumi et al. second area (16)	Japan	2016	333	56 (31-83)	45.2 ± 13.9	NA	31 (12-68)	144	48(31-72)	47.8 ± 13.1	NA	26 (14-57)	RCT
Park et al. (17)	South Korea	2016	21	15.2 ± 7.7	50.9 ± 8.4	NA	21.4 ± 39.7	11	14.5 ± 8.0	49.0 ± 6.5	NA	38.5 ± 48.8	RCT
Ko et al. (18)	South Korea	2017	1541	NA	42.6 ± 13	NA	18.6 ± 34.1	248	NA	44.2 ± 12.4	NA	19.6 ± 36.2	RCT
Song et al. (19)	South Korea	2017	121	41.1 ± 16.2	44.1 ± 12.5	NA	18.9 ± 39.1	95	38.0 ± 14.7	44.2 ± 13.54	NA	15.2 ± 30.7	RCT
Hamano et al. (20)	Japan	2020	70	74.4 ± 44.4	43.0 ± 14.0	NA	18.0 ± 36.0	37	61.2 ± 38.4	47.0 ± 13.0	NA	31.2 ± 69.6	RCT
Jiang et al. (8)	China	2020	779	NA	32.8 ± 5.7	22.2 ± 3.8	23.4 ± 16.7	342	NA	34.5 ± 7.4	24.6 ± 4.3	23.4 ± 16.7	RCT
Ko et al. (21)	South Korea	2020	1190	NA	45.9 ± 12.0	25.0 ± 7.5	20.9 ± 37.9	358	NA	47.3 ± 11.6	23.5 ± 9.1	20.2 ± 33.2	RCT
Wang et al. (22)	China	2020	96	29.8 (2-52)	29.0 (15-53)	20.2 (14.7-31.6)	12 (0-108)	48	27.6(4-51.6)	30 (9-53)	20.4 (14.8-32.6)	12 (0-96)	RCT
Kim et al. (23)	South Korea	2021	222	NA	63.9 ± 3.5	23.4 ± 3.1	3 (1-13)	80	NA	63.7 ± 3.2	24.9 ± 9.1	2 (1-17)	RCT
Prabhakar et al. (24)	India	2021	100	27.2 ± 20.6	40.8 (17-68)	NA	5.5 (0-12)	100	25.9 ± 20.5	40.9 (17-61)	NA	5.77 (0-12)	RCT

Data are presented as mean ± SD or median (IQR) as indicated in the table. Abbreviations: ABOi-LDKT, ABO-incompatible living donor kidney transplantation; ABOc-LDKT, ABO-compatible living donor kidney transplantation. RCT, randomized controlled trial.

**Figure 2:**
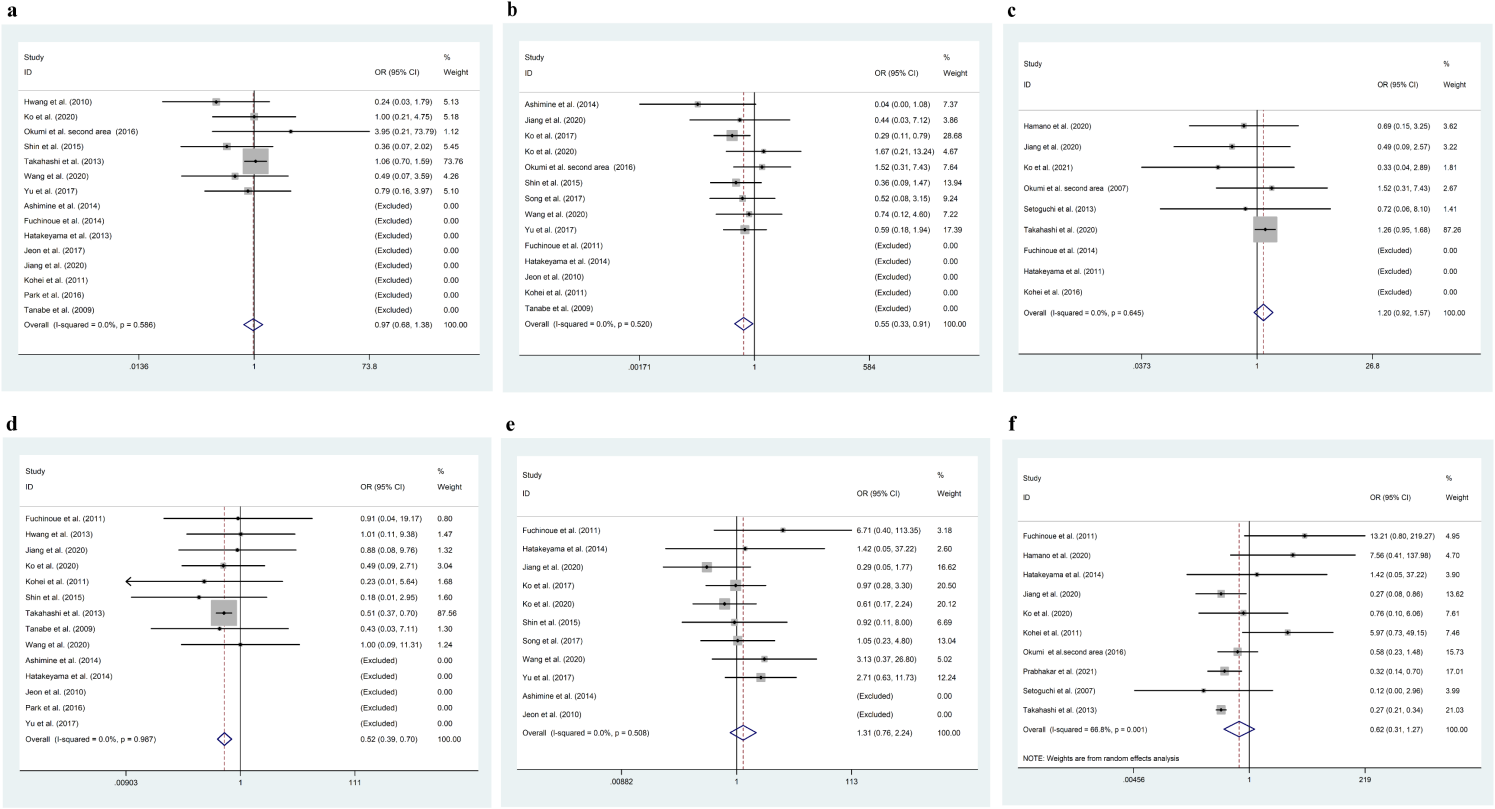
Meta-analysis results for patient and graft survival rates at 1, 3, and 5 years. **(a–c)** Patient survival rate for 1, 3, 5 years respectively. **(d–f)** Graft survival rate for 1, 3, 5 years respectively. Odds Ratios (ORs) with 95% confidence intervals (CIs) are shown. Significant differences are indicated where p < 0.05.

**Figure 3:**
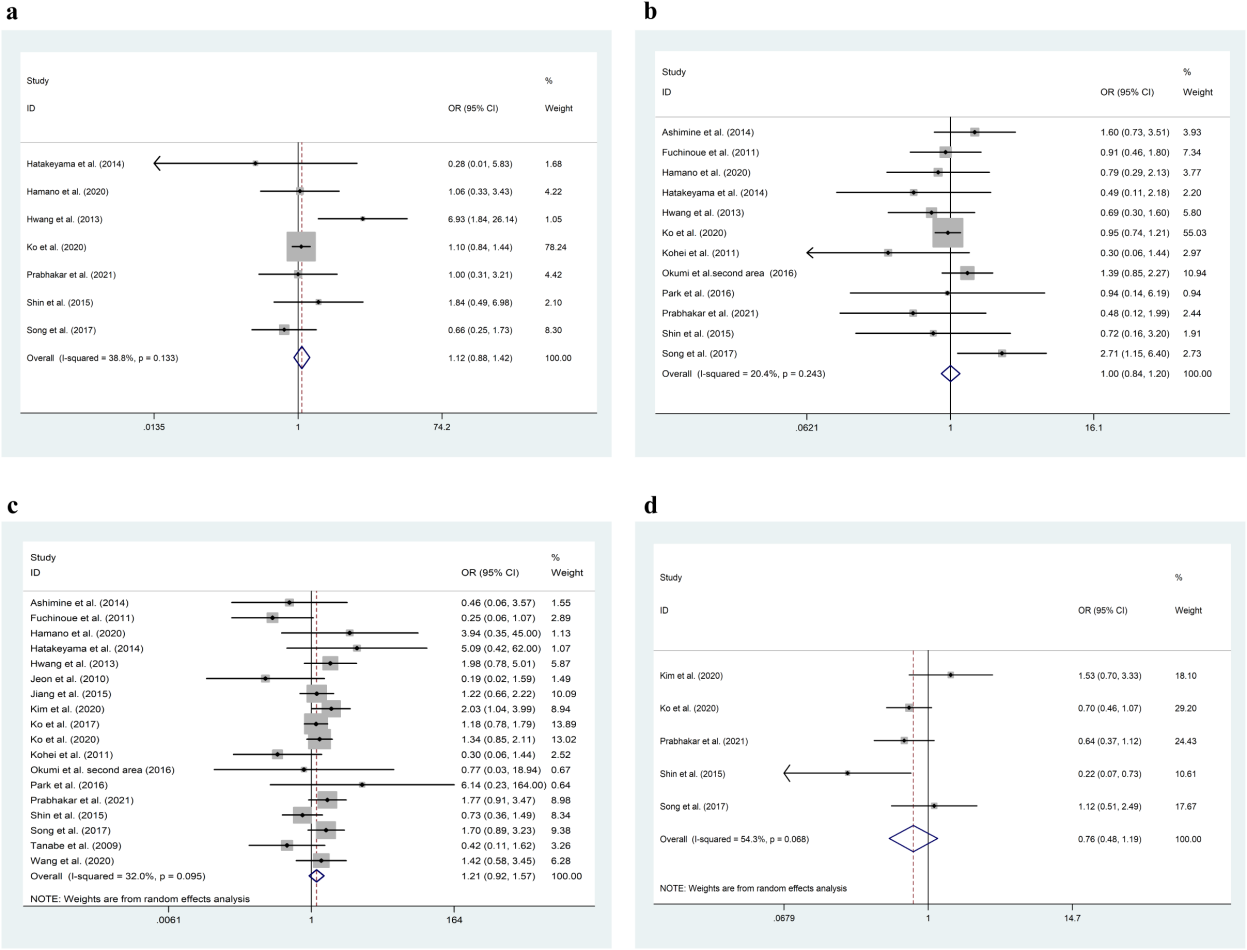
Subgroup analysis of the meta-analysis results. **(a)** BK virus-related outcomes. **(b)** CMV-related outcomes. **(c)** Acute rejection- related outcomes. **(d)** Urinary tract infection-related outcomes. Hazard ratios (HRs) with 95% confidence intervals (CIs) are shown. Significant differences are indicated where p < 0.05.

In the analysis of graft survival rates, 14 studies were included for the 1-year survival rate, 11 for the 3-year survival rate, and 10 for the 5-year survival rate. As shown in **Table 2** and **Figure 2**, the 1-year graft survival rate for the ABOi-LDKT group was lower than that for the ABOc-LDKT group (Pz < 0.01, OR = 0.518, CI = 0.386 - 0.695). However, there were no significant differences in the 3-year (Pz = 0.330, OR = 1.307, CI = 0.763 - 2.240) and 5-year (Pz = 0.193, OR = 0.623, CI = 0.305 - 1.270) survival rates between the two groups. In addition, the ABOi-LDKT group had a lower patient survival rate at 3 years (Pz = 0.021, OR = 0.552, CI = 0.333 - 0.914), but not at 1 year (Pz = 0.860, OR = 0.969, CI = 0.679 - 1.381) or 5 years (Pz = 0.187, OR = 1.199, CI = 0.916 - 1.569). In the subgroup analysis of postoperative complication risks, Cytomegalovirus (CMV) infection, BK virus infection, UTI, and postoperative acute rejection were investigated. No significant differences were observed between these groups, as shown by the forest plots in **Figure 3**.

**Table 2:** Meta-analysis results for patient survival rate, graft survival rate, and postoperative complications.

Outcome	Number of studies (N)	OR (95%Cl)	P_z_	P_H_	Model selection
Graft Survival Rate					
1-year	15	0.518 (0.386-0.695)	<0.01	0.987	Fixed
3-year	11	1.307 (0.763-2.240)	0.330	0.508	Fixed
5-year	10	0.623 (0.305-1.270)	0.193	0.001	Random
Patient Survival Rate					
1-year	15	0.969 (0.679-1.381)	0.860	0.586	Fixed
3-year	14	0.552 (0.333-0.914)	0.021	0.52	Fixed
5-year	9	1.199 (0.916-1.569)	0.187	0.645	Fixed
Complications					
Cytomegalovirus	13	1.002 (0.837-1.199)	0.982	0.243	Fixed
BK virus	7	1.121 (0.885-1.421)	0.344	0.133	Fixed
Urinary Infection	5	0.759 (0.483-1.194)	0.233	0.068	Random
Acute Rejection	18	1.207 (0.925-1.574)	0.166	0.095	Random

This table summarizes the pooled estimates for patient survival rates, graft survival rates, and postoperative complications based on data from included studies. Hazard ratios (HRs) with 95% confidence intervals (CIs) are reported for each time point.

Sensitivity analyses were performed using a leave-one-out strategy, sequentially excluding individual studies to evaluate the robustness of the pooled results. The recalculated pooled effect sizes (HRs/ORs) and corresponding 95% confidence intervals (CIs) remained consistent, confirming that no single study disproportionately influenced the overall findings (**Supplementary Table S1**; **Supplementary Figure S1**). Furthermore, publication bias was assessed through visual inspection of funnel plots and Begg’s rank correlation test. As shown in **Supplementary Figure S2** and **Supplementary Table S2**, the symmetrical distribution of the funnel plots, corroborated by the non-significant results of Begg’s test (P > 0.05), indicates the absence of substantial publication bias among the included studies.

### 3.2 Clinical study result

#### 3.2.1 Baseline characteristics of patients in the two groups

From January 1, 2021, to December 31, 2022, a total of 188 living donor kidney transplant recipients were screened at our center. In accordance with the prespecified eligibility criteria, 15 recipients who received grafts in the left iliac fossa were excluded to maintain technical homogeneity. The final cohort comprised 173 patients, including 41 in the ABOi-LDKT group and 132 in the ABOc-LDKT group. The screening process is summarized in **Figure 1b**. There were no statistically significant differences in sex, age, height, weight, body mass index (BMI), or donor GFR between the two donor and recipient groups. Baseline characteristics are summarized in **Table 3** and **Supplementary Figure S3**.

**Table 3:** The basic information of donors and recipients in ABOi-LDKT and ABOc-LDKT.

Characteristics	ABOi-LDKT (n = 41)	ABOc-LDKT (n = 132)	P
Doner gender (n)			0.683
Male	9	35	
Female	32	97	
Doner age (y)	57.07 ± 7.63	56.73 ± 7.03	0.754
Doner BMI (kg/m^2^)	24.47 ± 2.88	24.17 ± 3.55	0.447
Donor kidney GFR by renography (mL/min)	36.25 ± 9.60	35.26 ± 8.12	0.565
Recipients Gender (n)			0.301
Male	28	102	
Female	13	30	
Recipient age (y)	33.61 ± 7.57	34.55 ± 12.87	0.974
Recipient BMI (kg/m^2^)	21.13 ± 2.81	22.16 ± 3.65	0.064
Preoperative Hypertension Status (n)	27	66	0.106
Preoperative Hypertension Duration (mo)	49.44 ± 52.21	33.41 ± 30.73	0.237
Preoperative Dialysis Status (n)			0.550
Hemodialysis	28	89	
Peritoneal Dialysis	5	24	
None	8	19	
Preoperative Dialysis Duration (mo)	25.61 ± 27.25	24.81 ± 31.25	0.796

Donor kidney GFR by renography: measured preoperatively by renal dynamic scintigraphy with ^99mTc-DTPA using the Gates camera method; values are reported as mL/min and represent the whole-kidney GFR of the donated side. Time units: durations are expressed in months (mo) unless otherwise indicated; years (y) are used where specified. Data are presented as mean ± SD as indicated in the table. Abbreviations: BMI, Body Mass Index, values are reported as kg/m^2^; ABOi-LDKT, ABO-incompatible living donor kidney transplantation; ABOc-LDKT, ABO-compatible living donor kidney transplantation.

#### 3.2.2 Clinical data analysis results of the two groups of patients

**Figure 4:**
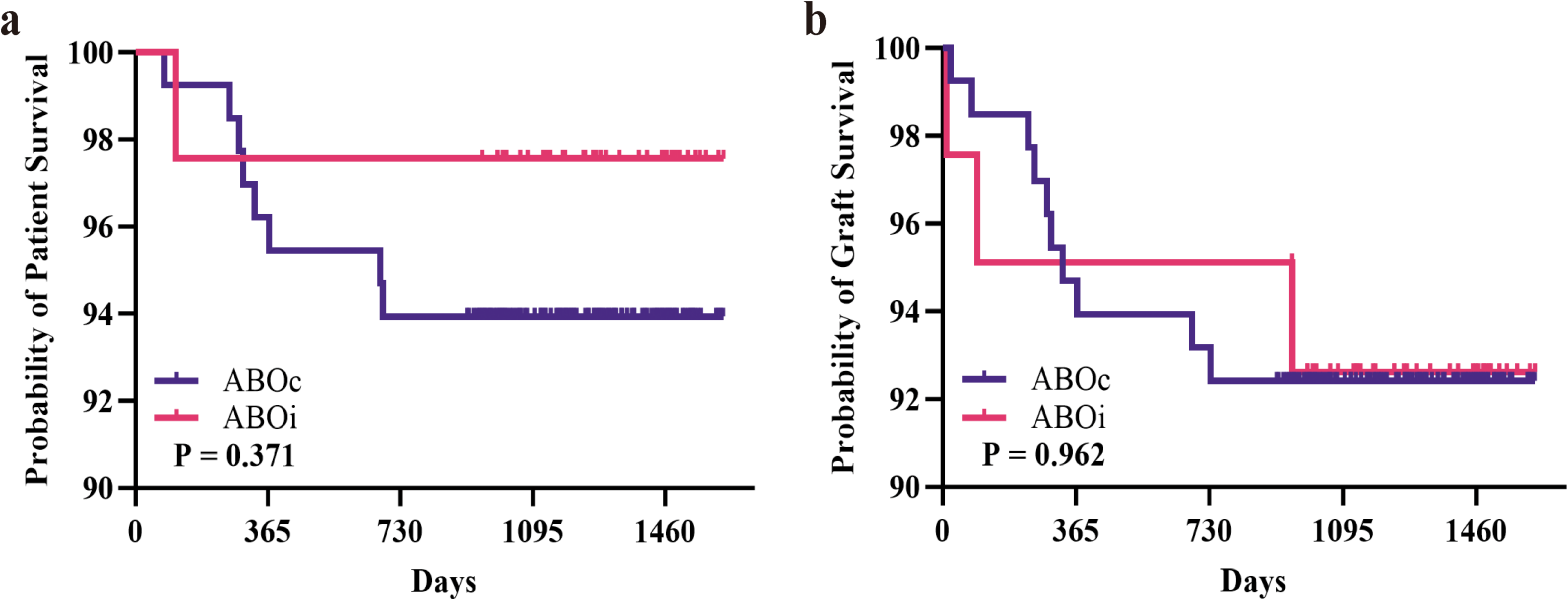
Survival outcomes of ABOi-LDKT and ABOc-LDKT groups. **(a)** Kaplan–Meier survival curves for patient survival in the two groups. **(b)** Kaplan–Meier survival curves for graft survival in the two groups.

**Figure 5:**
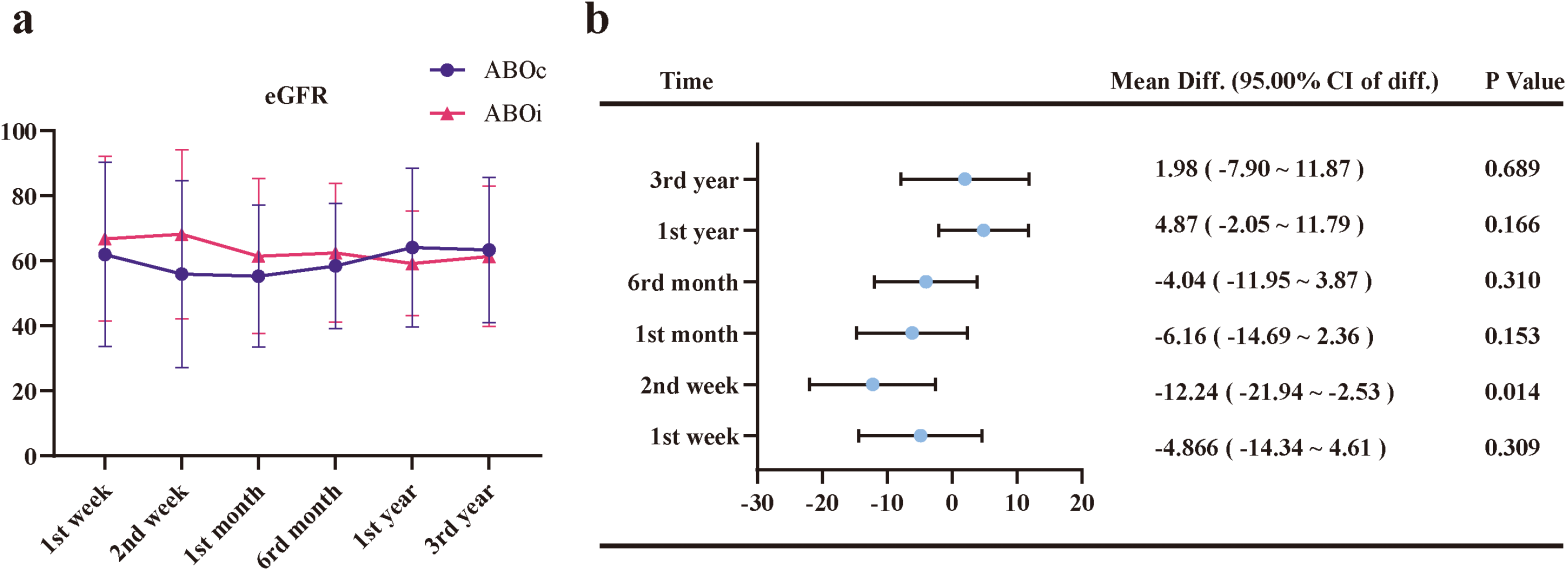
Longitudinal changes in estimated glomerular filtration rate (eGFR) and corresponding mean differences between the ABOi and ABOc groups. **(a)** Trends in eGFR levels at 1 week, 2 weeks, 1 month, 6 months, 1 year, and 3 years post-transplantation. **(b)** Forest plot showing mean differences (Mean Diff.) and 95.00% confidence intervals of the difference (95.00% CI of diff.) for eGFR between the ABOi and ABOc groups at each time point. Error bars represent standard deviations. A statistically significant difference was observed at 2 weeks post-transplantation (p < 0.05), while no significant differences were found at other time points.

All patients were followed until June 1, 2025. No significant differences were observed in patient or graft survival at 1 and 3 years between the ABOi-LDKT and ABOc-LDKT groups (**Figures 4a, b** and **Table 4**). Longitudinal renal function assessment revealed that ABOi recipients achieved superior eGFR at 2 weeks post-transplantation (P<0.05), likely attributable to the preconditioning apheresis. However, this difference was transient, with no significant disparities in eGFR or Scr levels observed at 1 month, 6 months, 1 year, or 3 years (**Figure 5**; **Supplementary Table S3**). Consistent with these findings, the analysis of SCr levels demonstrated similar recovery trajectories between the two groups, with no significant differences observed during the long-term follow-up period (**Supplementary Figure S4**; **Supplementary Table S3**).

**Table 4:** The clinical data analysis results of the two groups of patients.

Clinical Outcomes	ABOi-LDKT	ABOc-LDKT	P
Number	41	132	
Postoperative Hospital Stay (Day)	22.27 ± 7.10	22.20 ± 9.30	0.955
In-patient Care Spending (thousand CNY)	127.13 ± 41.21	91.34 ± 38.74	<0.001
Delayed Graft Function	2.44%	3.03%	1.000
Pulmonary Infection	34.15%	20.45%	0.092
Urinary Tract Infection	4.88%	3.79%	0.670
Surgical Complication	14.63%	8.33%	0.240
Grafts Survival 1^st^ year	95.12%	94.70%	1.000
Grafts Survival 3^rd^ year	92.68%	92.42%	1.000
Patients Survival 1^st^ year	97.56%	96.21%	1.000
Patients Survival 3^rd^ year	97.56%	93.94%	0.692

Infection data represent episodes of severe infection requiring re-hospitalization within 1 year post-transplantation.

AMR occurred in only one patient (2.4%) in the ABOi-LDKT group on postoperative day 7. This patient presented with oliguria and a rebound in isoagglutinin titers but responded well to rescue therapy (PE and pulse steroids) with full functional recovery. The absence of rejection in the ABOc group and the rapid reversal of the single AMR case in the ABOi group underscore the efficacy of the immunosuppression protocols employed.

There were no statistically significant differences in the incidence of surgical complications (14.6% vs. 8.3%, P = 0.240). Specific complications in the ABOi group included ureteral stenosis (n=1), perinephric effusion (n=1), anastomotic bleeding (n=1), and wound dehiscence (n=3). All patients were successfully discharged following active treatment. Infection surveillance showed a numerically higher rate of pulmonary infection in the ABOi group (34.1% vs. 20.5%) and comparable rates of UTI (4.9% vs. 3.8%), though neither reached statistical significance. This relatively high incidence of pulmonary infection may be partly attributed to the overlap of the study period with the COVID-19 pandemic, as well as the strict criterion of recording only infections necessitating re-hospitalization. While the length of postoperative hospital stay was similar between groups (P = 0.955), total hospitalization costs were significantly higher for ABOi recipients (P<0.001), reflecting the additional resource utilization associated with desensitization therapy (**Table 4**).

#### 3.2.3 Immunological outcomes and regimen efficacy

**Figure 6:**
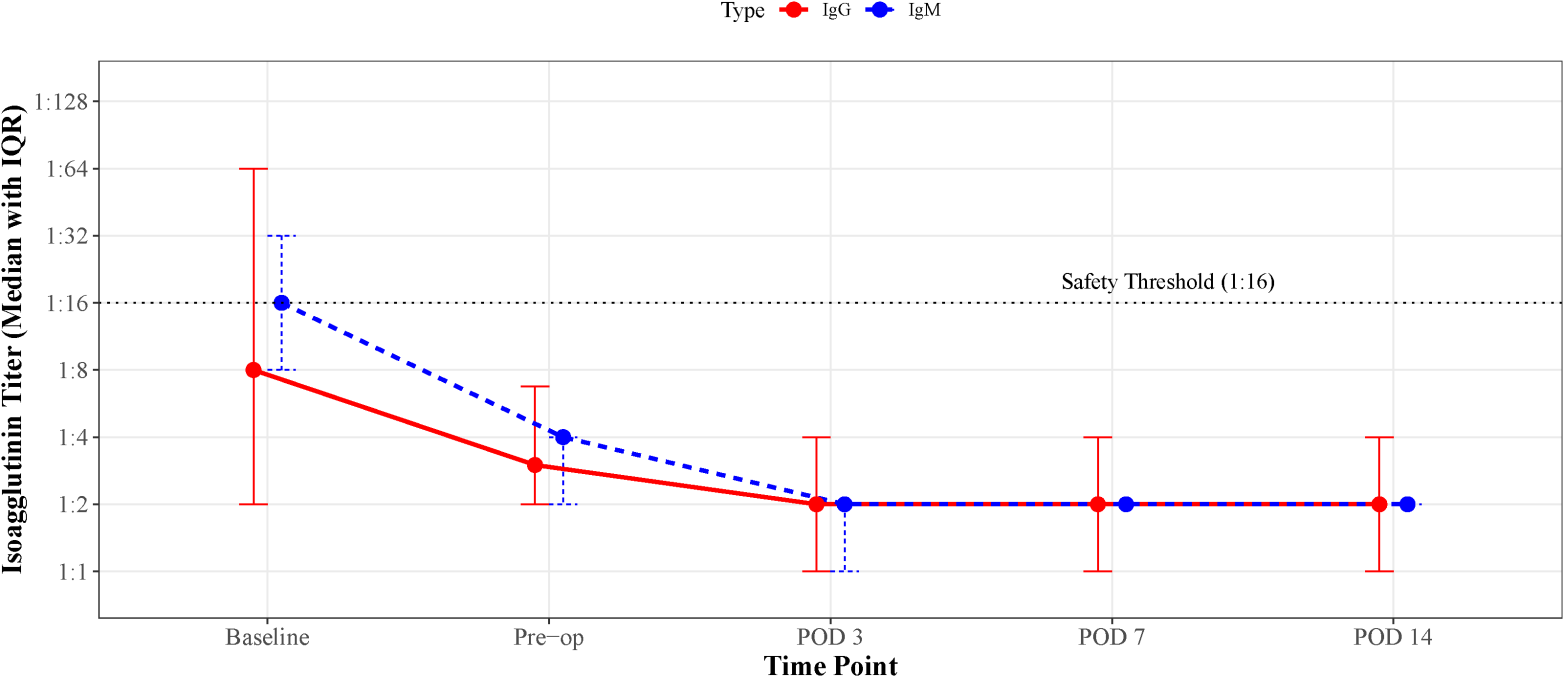
Effectiveness of the desensitization protocol and longitudinal dynamics of isoagglutinin titers. The graph illustrates the longitudinal changes in median IgM (blue solid line) and IgG (red dashed line) isoagglutinin titers for the ABOi cohort (n=41) at five key time points: baseline, pre-operation (Pre-op), and postoperative days (POD) 3, 7, and 14. Data points represent the median titer, and error bars represent the interquartile range (IQR). To ensure visual clarity, data points for IgM and IgG at the same time intervals are slightly offset. The horizontal dotted line at 1:16 indicates the clinical safety threshold for transplantation. Following the individualized desensitization regimen, median titers for both IgM and IgG significantly declined from baseline to pre-operation (P<0.001) and remained consistently low (≤1:2) throughout the early postoperative period, demonstrating effective antibody depletion and the absence of significant rebound.

All 41 ABOi recipients underwent an individualized desensitization regimen tailored to their baseline immunological risk. The median number of pre-conditioning PE/DFPP sessions required was 2 (range: 1–12). This protocol demonstrated robust effectiveness: the median baseline isoagglutinin titers were 1:16 (IQR 1:8–1:32) for IgM and 1:8 (IQR 1:2–1:64) for IgG. Immediately prior to transplantation, median titers were significantly reduced to 1:4 (IQR 1:2–1:4) for IgM and 1:4 (IQR 1:2–1:8) for IgG (P<0.001), with 100% of patients achieving the target titer of ≤1:16. Postoperatively, antibody levels remained stable; the median titers for both IgM and IgG at postoperative days 3, 7, and 14 maintained at low levels (≤1:2), as shown in **Figure 6**, indicating minimal antibody rebound.

## 4 Discussion

Despite the established status of ABOi-LDKT globally, its application in Asian populations has been hindered by concerns regarding the balance between aggressive desensitization and the risks of infection or rejection. This study aimed to comprehensively evaluate the “viability” of ABOi-LDKT in the Chinese setting through a dual approach: a systematic meta-analysis of Asian cohorts and a rigorous single-center validation. Our meta-analysis revealed that while ABOi-LDKT carries a historically higher risk of early graft loss and patient mortality compared to ABOc-LDKT transplants, our single-center experience demonstrates that these risks can be effectively mitigated. By implementing an individualized Rituximab-based desensitization protocol and stratified infection prophylaxis, we achieved 1-year and 3-year patient and graft survival rates comparable to ABOc-LDKT. These findings suggest that the “viability” of ABOi-LDKT is not an inherent fixity but is contingent upon the effectiveness of antibody depletion, the safety of perioperative management, and the acceptance of its economic trade-offs.

The primary determinant of ABOi-LDKT effectiveness is the successful depletion of isoagglutinins to prevent hyperacute rejection while fostering immunological accommodation. Historically, splenectomy was the standard for B-cell reduction, but it carried significant morbidity (6, 13). Our study validates the efficacy of a contemporary, spleen-preserving regimen utilizing low-dose Rituximab tailored to preoperative CD19^+^ B-cell counts. This “individualized” strategy proved highly effective: All of ABOi recipients maintained isoagglutinin titers below the safety threshold of 1:16 without needing rescue splenectomy. Notably, our single-center cohort reported a very low incidence of biopsy-proven acute rejection, with only one case (2.4%) of AMR. This case, characterized by a transient rebound of titers, was successfully reversed with plasma exchange, supporting the theory of “accommodation”—whereby the graft develops resistance to humoral injury over time despite the recurrence of antibodies (25). Furthermore, the observation that ABOi recipients exhibited superior eGFR at 2 weeks post-transplantation suggests that effective pre-conditioning, particularly PE, may remove inflammatory cytokines and mitigate early ischemia-reperfusion injury. Thus, our data confirm that modern desensitization protocols can effectively overcome the immunological barrier of blood type incompatibility.

Safety concerns, particularly infection-related mortality, have long been the Achilles’ heel of ABOi-LDKT. Our meta-analysis indicated significantly lower 3-year patient survival in pooled Asian cohorts, a finding consistent with USRDS data where infection remains a leading cause of death in desensitized patients (26, 27). However, our single-center results diverge positively from this trend, showing no significant difference in survival or severe infectious complications between ABOi and ABOc groups. We attribute this safety profile to our “Standardized Perioperative Management” protocol. Unlike earlier eras where “blanket” immunosuppression was common, we employed a risk-stratified approach. Specifically, Ganciclovir dosing for CMV prophylaxis was strictly adjusted based on postoperative eGFR (5-tier stratification), and Sulfamethoxazole-Trimethoprim was routinely initiated for *Pneumocystis* pneumonia prophylaxis. Although the incidence of pulmonary infection was numerically higher in the ABOi group (34.1% vs. 20.5%), reflecting the inevitable impact of enhanced immunosuppression, the lack of statistical significance and the absence of infection-related mortality highlight that these risks are manageable. This underscores that the safety of ABOi-LDKT relies less on reducing immunosuppression intensity and more on vigilant surveillance and preemptive antimicrobial strategy.

In the context of developing nations, the economic viability of ABOi-LDKT is as critical as its clinical success. Critics often point to the high upfront costs as a barrier. Indeed, our analysis confirmed that hospitalization costs for ABOi-LDKT were significantly higher ($127.1k vs. $91.3k CNY), driven largely by the specialized requirements of PE, adsorbers, and desensitization pharmacotherapy (28). However, this “sticker shock” must be contextualized within the broader health-economic landscape. The cost of maintaining a patient on hemodialysis for 2–3 years frequently exceeds the one-time premium of ABOi transplantation, not to mention the profound disparity in quality of life and societal reintegration (3, 29). Therefore, while ABOi-LDKT presents a higher initial financial threshold, it remains a cost-effective long-term investment for healthcare systems and a life-altering opportunity for patients stranded on waitlists.

This study has limitations inherent to its design. The single-center retrospective nature and modest sample size of the ABOi cohort precluded the use of propensity score matching, potentially introducing selection bias. Additionally, while we focused on short- to mid-term outcomes (up to 3 years), long-term complications such as chronic antibody-mediated rejection and late graft fibrosis require extended follow-up to fully assess. Finally, the heterogeneity of desensitization protocols in the included meta-analysis studies may affect the generalizability of the pooled risk estimates.

## 5 Conclusion

In conclusion, by integrating a meta-analysis of Asian cohorts with single-center clinical validation, this study confirms the viability of ABOi-LDKT for Chinese patients. Within the constraints of sample size and follow-up duration, we demonstrated that an individualized desensitization protocol combined with stratified infection prophylaxis can achieve 1-year and 3-year patient and graft survival rates comparable to ABOc-LDKT. Although ABOi-LDKT incurs higher upfront hospitalization costs, the effective depletion of isoagglutinins successfully minimized early acute rejection risks and supported favorable graft function recovery without increasing severe infectious complications. These findings provide robust, context-specific evidence that ABOi-LDKT is a clinically feasible and safe strategy to expand the living donor pool in China. Future large-scale, multicenter studies with extended follow-up are warranted to validate long-term outcomes and further optimize the cost-effectiveness of these protocols.

## Data Availability

The original contributions presented in the study are included in the article/**Supplementary Material**. Further inquiries can be directed to the corresponding authors.

## Supplementary material

Supplementary Figure 1 Sensitivity analysis results from the meta-analysis. **(a)** Patient survival rate at 1st year post-transplantation. **(b)** Graft survival rate at 1st year post-transplantation. **(c)** Patient survival rate at 3rd year post-transplantation. **(d)** Graft survival rate at 3rd year post-transplantation. **(e)** Patient survival rate at 5th year post-transplantation. **(f)** Graft survival rate at 5th year post-transplantation. **(g)** Outcomes related to BK virus infection. **(h)** Outcomes related to Cytomegalovirus (CMV) infection. **(i)** Outcomes related to acute rejection. **(j)** Outcomes related to urinary tract infection (UTI). Each panel presents the results of the sensitivity analysis for the corresponding outcome, showing pooled hazard ratios (HRs) with 95% confidence intervals (CIs). Significant differences are indicated where p < 0.05.

Supplementary Figure 2 Funnel plot results from the meta-analysis. **(a)** Patient survival rate at 1st year post-transplantation. **(b)** Graft survival rate at 1st year post-transplantation. **(c)** Patient survival rate at 3rd year post-transplantation. **(d)** Graft survival rate at 3rd year post-transplantation. **(e)** Patient survival rate at 5th year post-transplantation. **(f)** Graft survival rate at 5th year post-transplantation. **(g)** BK virus infection outcomes. **(h)** Cytomegalovirus (CMV) infection outcomes. **(i)** Acute rejection outcomes. **(j)** Urinary tract infection (UTI) outcomes. Each panel represents a funnel plot showing the distribution of effect sizes for the corresponding outcome. The symmetry of the plots suggests the absence of publication bias. Significant differences are indicated where p < 0.05.

Supplementary Figure 3 Baseline demographic and clinical characteristics of donors and recipients in the ABOi-LDKT and ABOc-LDKT groups.

Supplementary Figure 4 Longitudinal changes in serum creatinine (Scr) levels and corresponding mean differences between the ABOi and ABOc groups. **(a)** Trends in Scr levels at 1 week, 2 weeks, 1 month, 6 months, 1 year, and 3 years post-transplantation. **(b)** Forest plot showing mean differences (Mean Diff.) and 95.00% confidence intervals of the difference (95.00% CI of diff.) for Scr between the ABOi and ABOc groups at each time point. Error bars represent standard deviations. Consistent with the eGFR findings, no clinically significant long-term differences were observed between the two groups.
