# Supplementary figures and images for: The viability of ABO-incompatible kidney transplants: a single-center cohort in China
Source: Front Immunol. 2026 Feb 17;17:1747411. doi: 10.3389/fimmu.2026.1747411 (PMC12953097; doi:10.3389/fimmu.2026.1747411)

**a**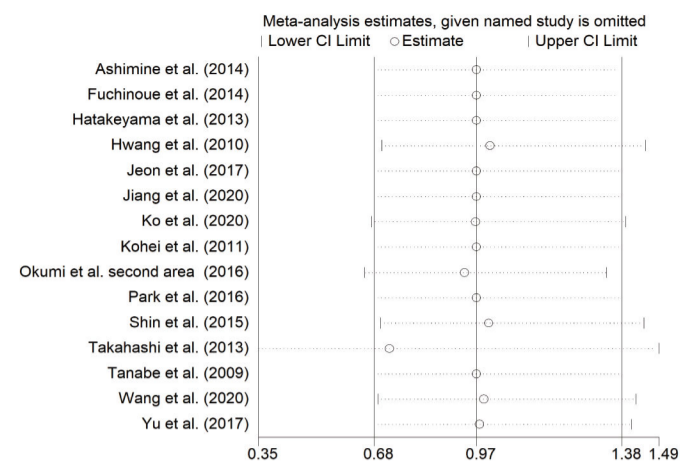**b**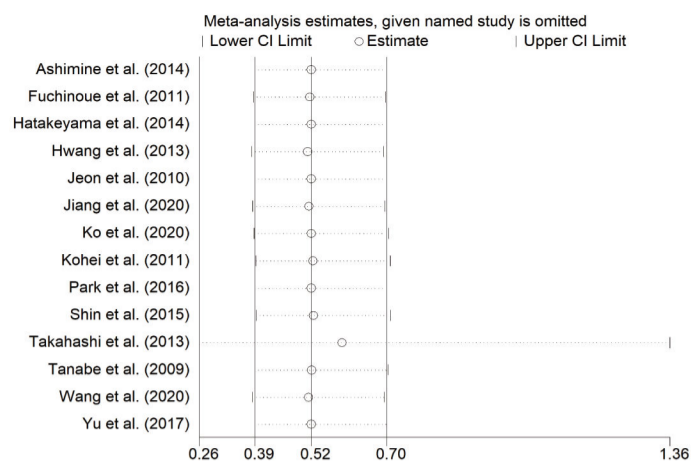**c**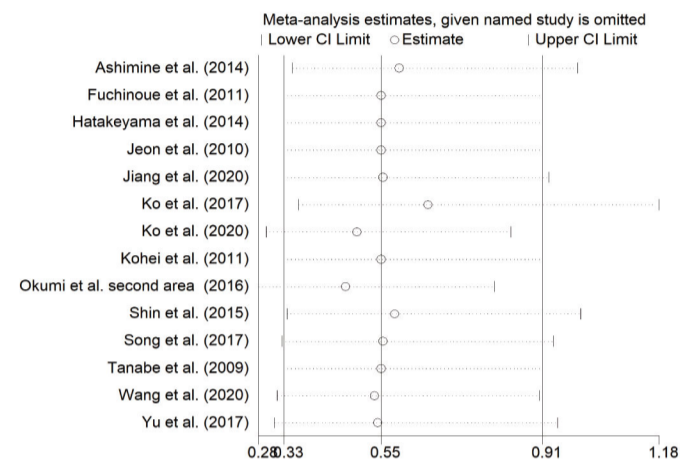**d**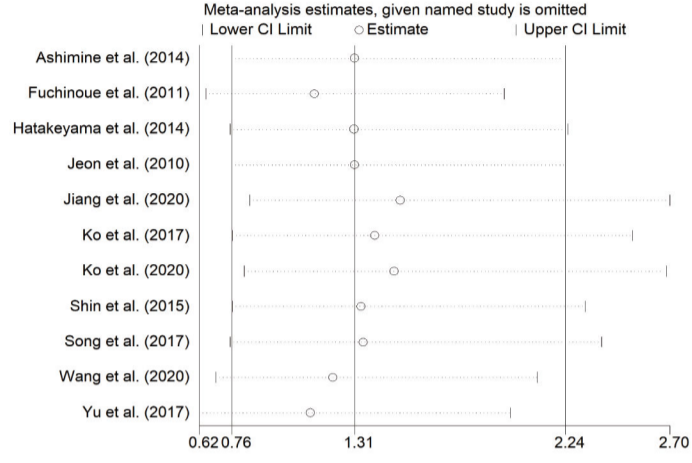**e**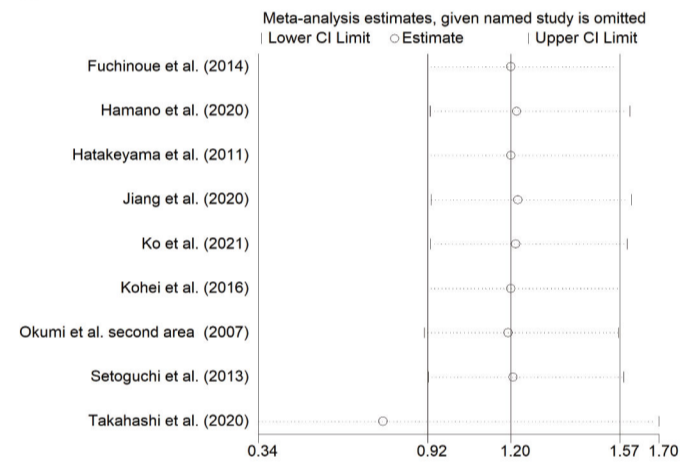**f**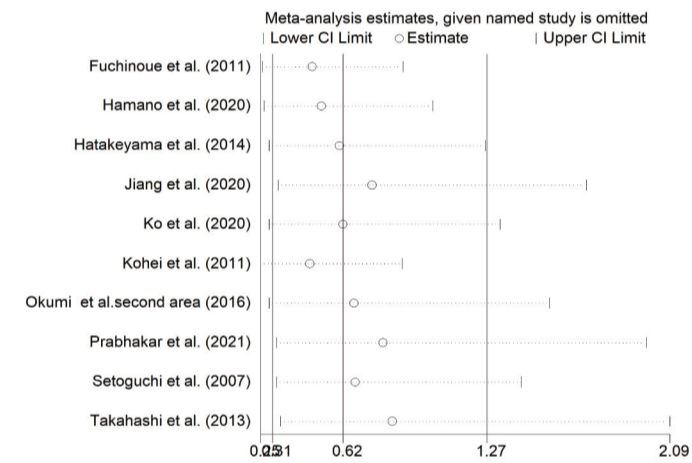**g**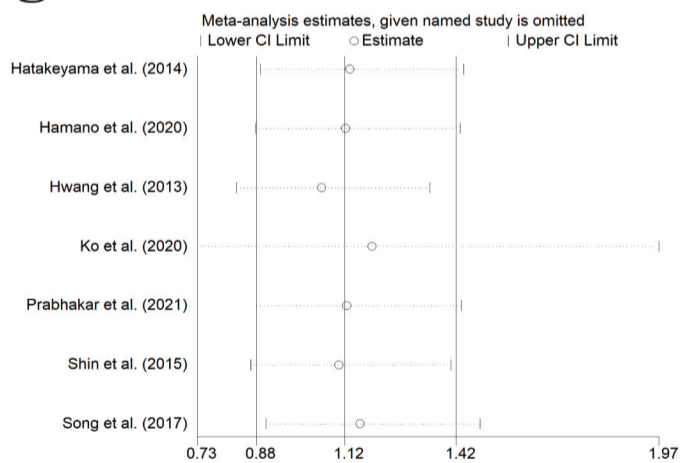**h**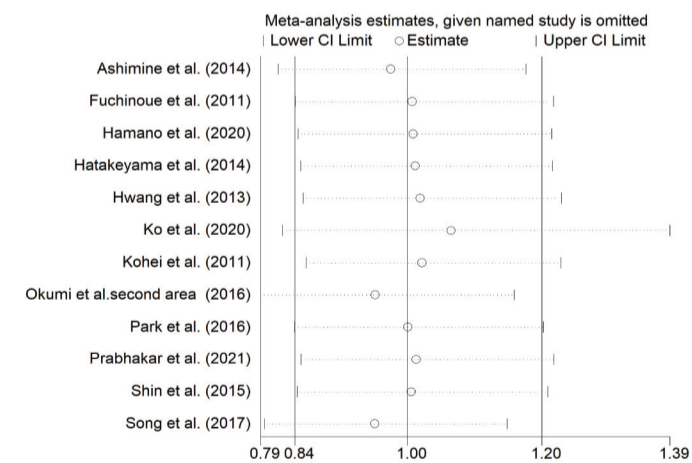**i**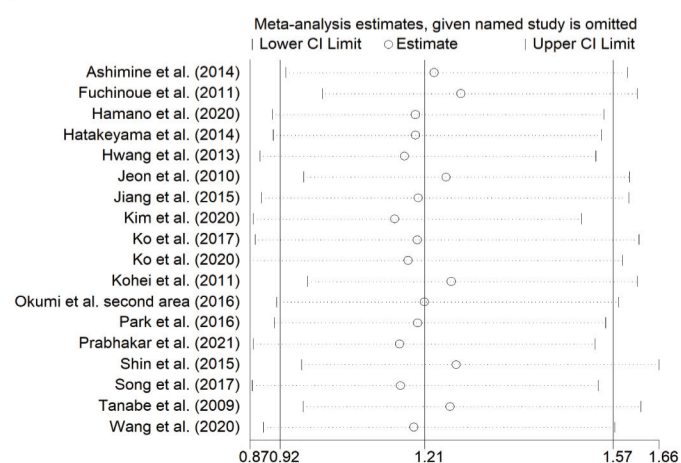**j**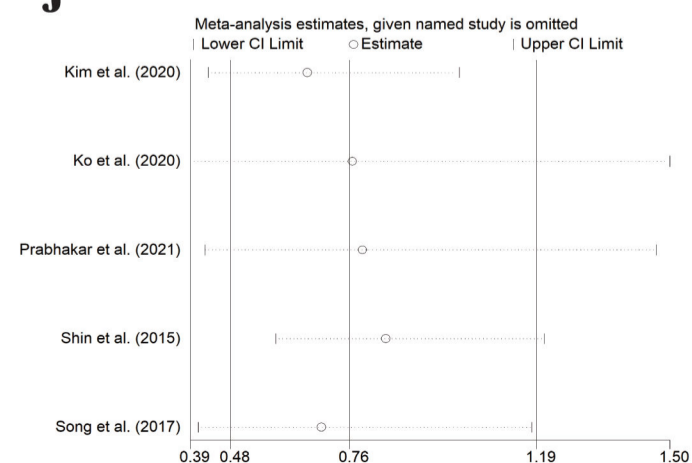

Supplement: Supplementary Figure 1 — Sensitivity analysis results from the meta-analysis. (a) Patient survival rate at 1st year post-transplantation. (b) Graft survival rate at 1st year post-transplantation. (c) Patient survival rate at 3rd year post-transplantation. (d) Graft survival rate at 3rd year post-transplantation. (e) Patient survival rate at 5th year post-transplantation. (f) Graft survival rate at 5th year post-transplantation. (g) Outcomes related to BK virus infection. (h) Outcomes related to Cytomegalovirus (CMV) infection. (i) Outcomes related to acute rejection. (j) Outcomes related to urinary tract infection (UTI). Each panel presents the results of the sensitivity analysis for the corresponding outcome, showing pooled hazard ratios (HRs) with 95% confidence intervals (CIs). Significant differences are indicated where p < 0.05. [file Image1.pdf]

**a**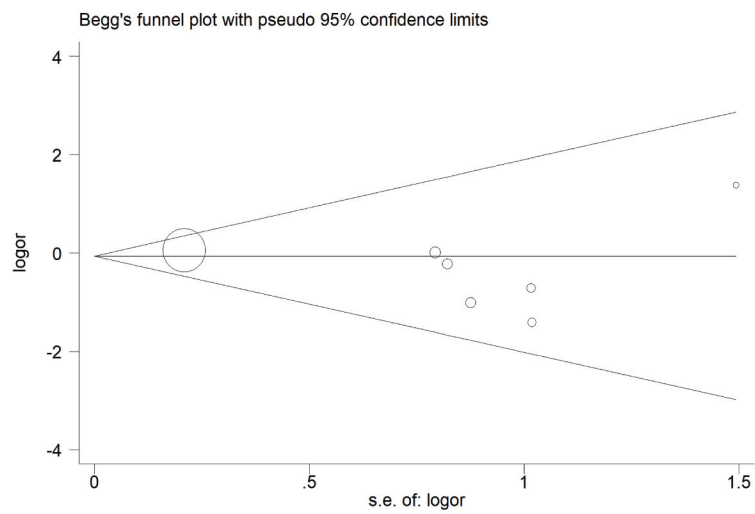**b**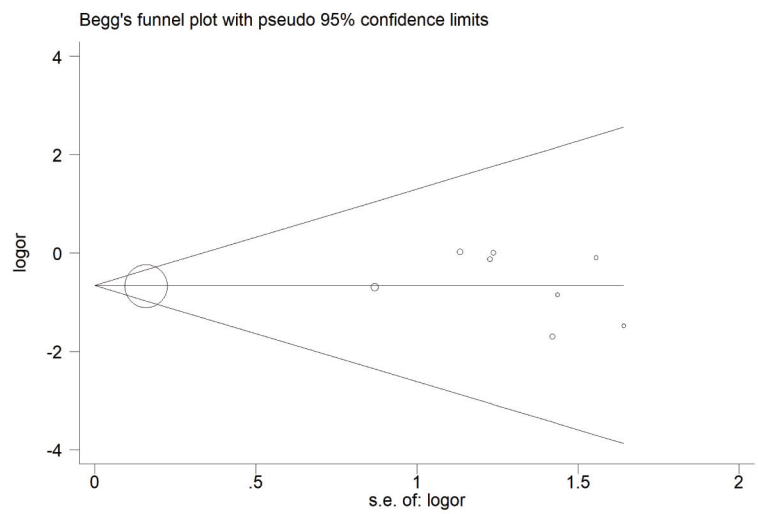**c**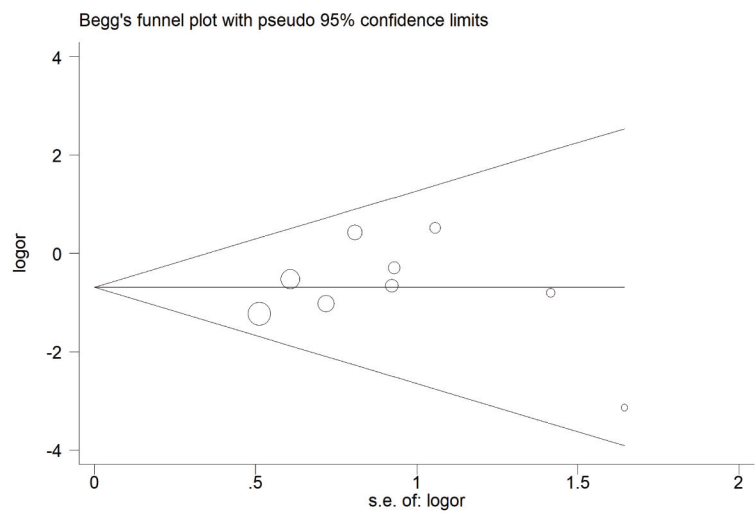**d**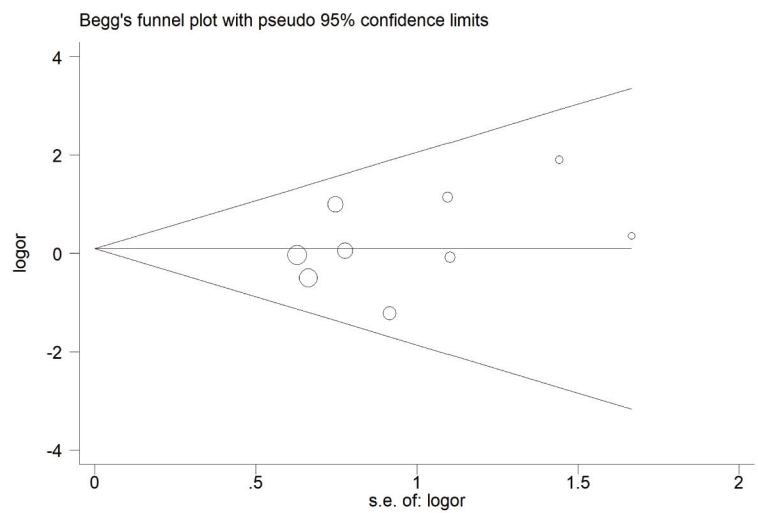**e**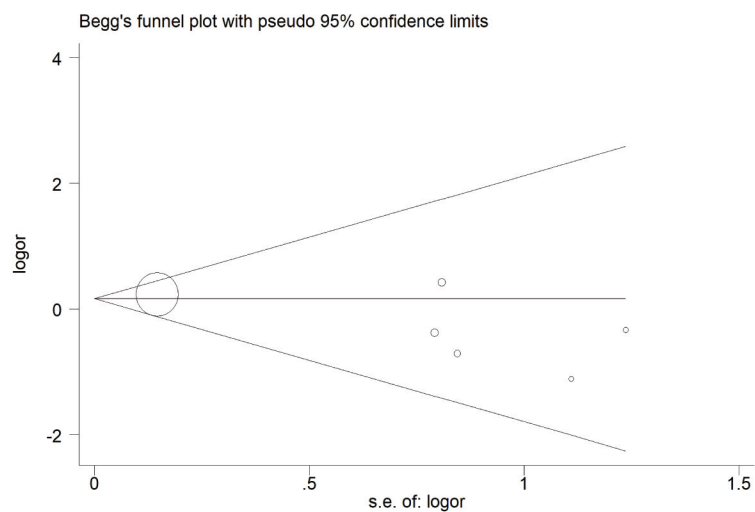**f**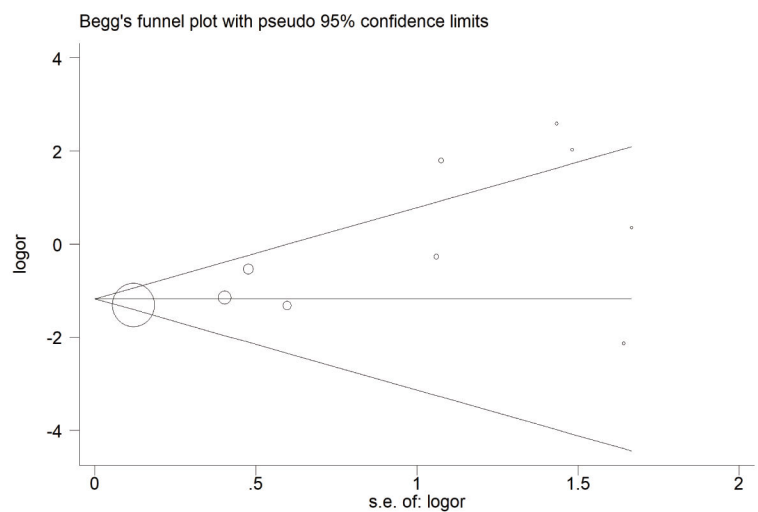**g**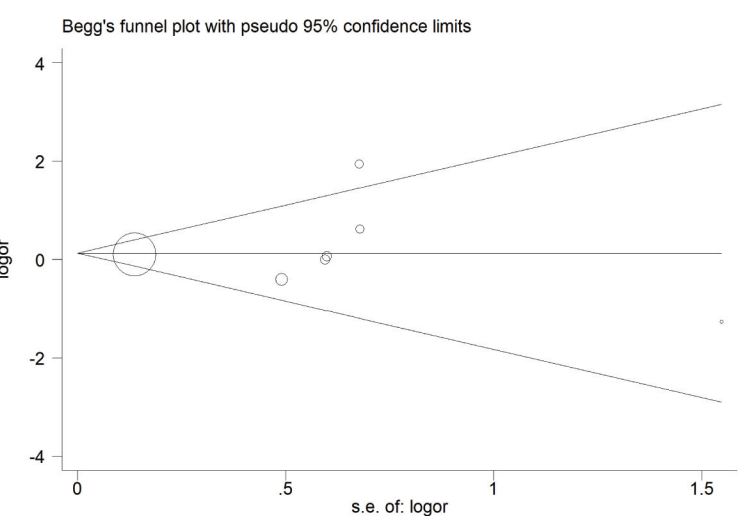**h**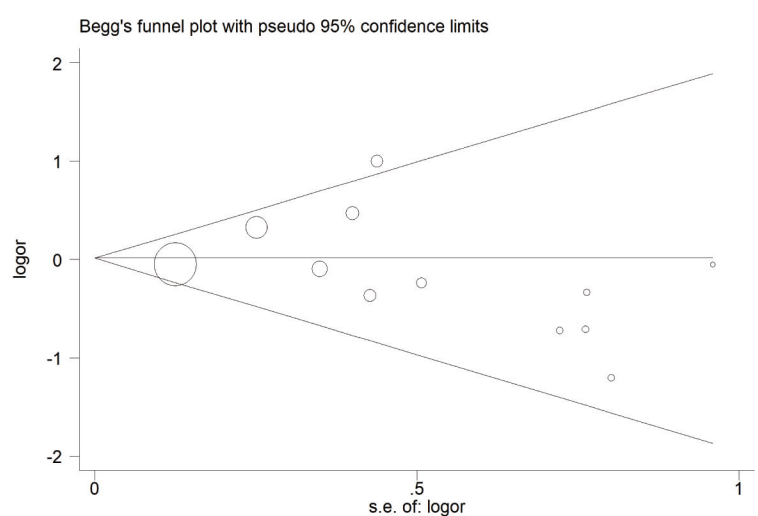**i**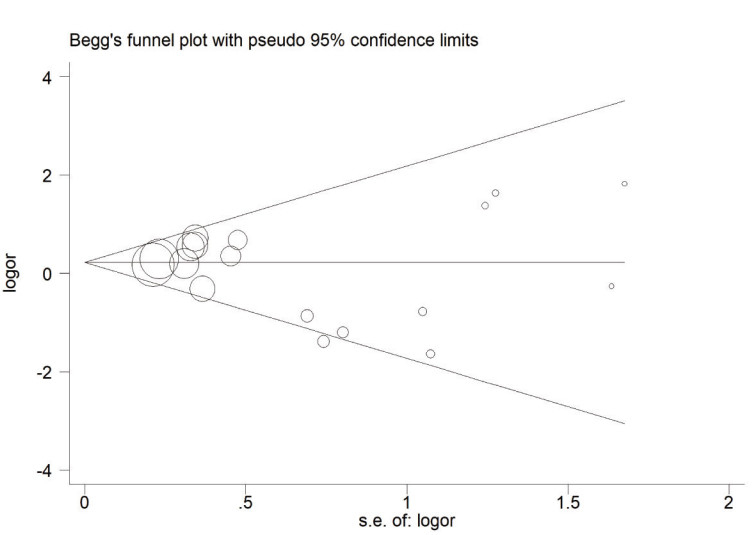**j**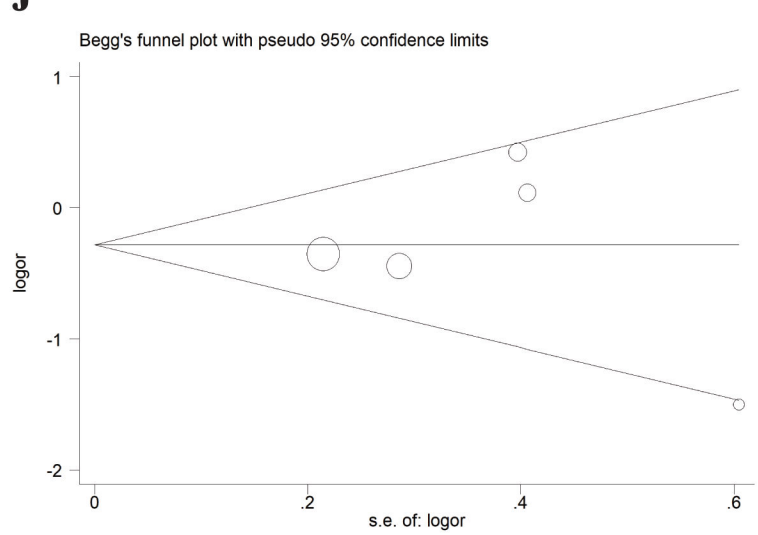

Supplement: Supplementary Figure 2 — Funnel plot results from the meta-analysis. (a) Patient survival rate at 1st year post-transplantation. (b) Graft survival rate at 1st year post-transplantation. (c) Patient survival rate at 3rd year post-transplantation. (d) Graft survival rate at 3rd year post-transplantation. (e) Patient survival rate at 5th year post-transplantation. (f) Graft survival rate at 5th year post-transplantation. (g) BK virus infection outcomes. (h) Cytomegalovirus (CMV) infection outcomes. (i) Acute rejection outcomes. (j) Urinary tract infection (UTI) outcomes. Each panel represents a funnel plot showing the distribution of effect sizes for the corresponding outcome. The symmetry of the plots suggests the absence of publication bias. Significant differences are indicated where p < 0.05. [file Image2.pdf]

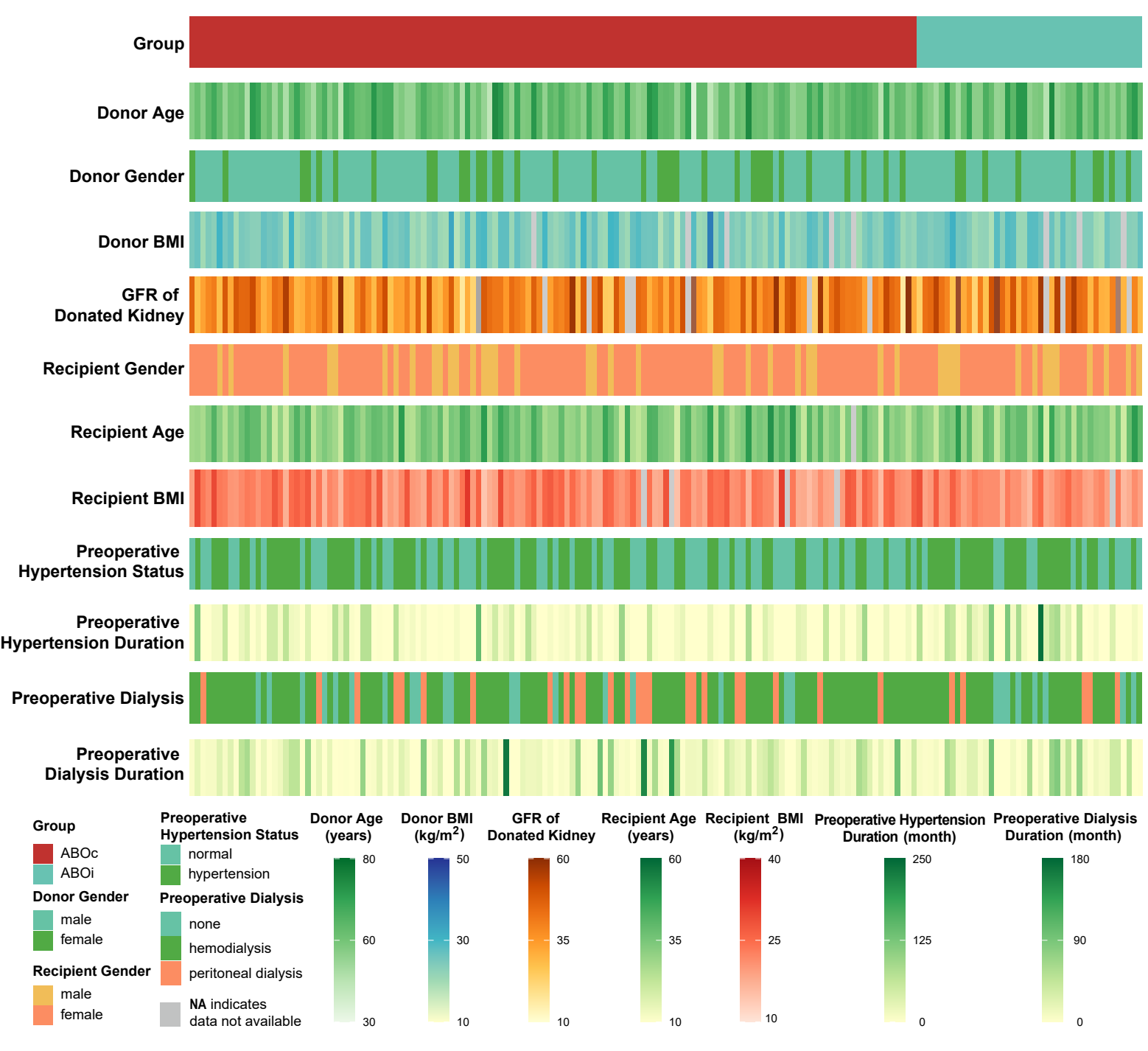

Supplement: Supplementary Figure 3 — Baseline demographic and clinical characteristics of donors and recipients in the ABOi-LDKT and ABOc-LDKT groups. [file Image3.pdf]

**a**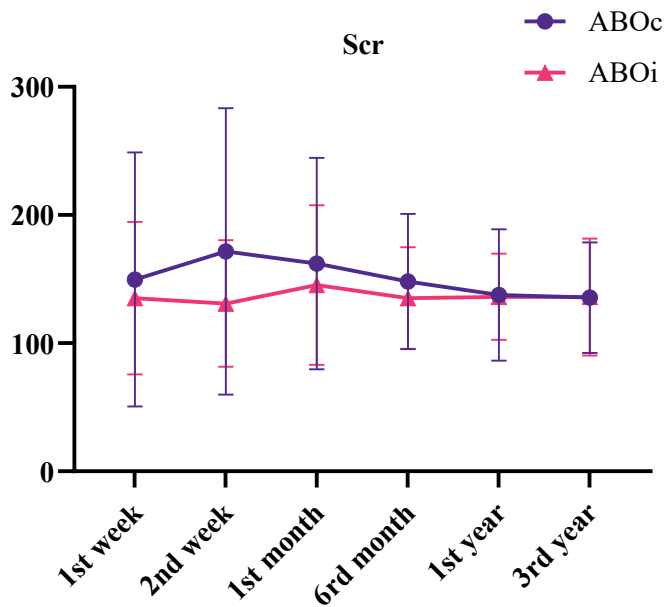**b**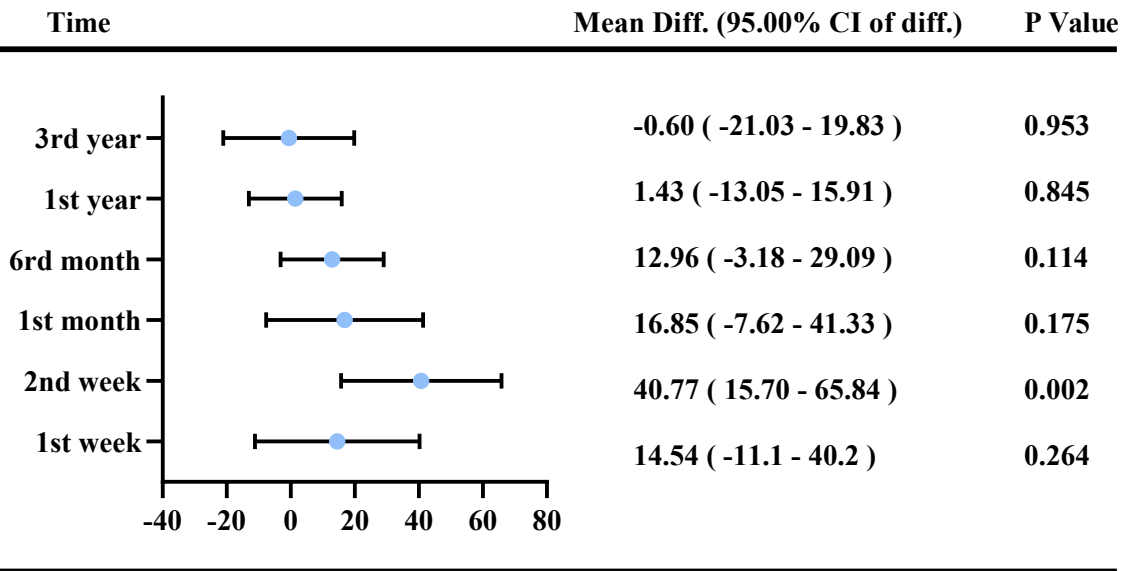

Supplement: Supplementary Figure 4 — Longitudinal changes in serum creatinine (Scr) levels and corresponding mean differences between the ABOi and ABOc groups. (a) Trends in Scr levels at 1 week, 2 weeks, 1 month, 6 months, 1 year, and 3 years post-transplantation. (b) Forest plot showing mean differences (Mean Diff.) and 95.00% confidence intervals of the difference (95.00% CI of diff.) for Scr between the ABOi and ABOc groups at each time point. Error bars represent standard deviations. Consistent with the eGFR findings, no clinically significant long-term differences were observed between the two groups. [file Image4.pdf]
