# Supplementary material for: The viability of ABO-incompatible kidney transplants: a single-center cohort in China
Source: Front Immunol. 2026 Feb 17;17:1747411. doi: 10.3389/fimmu.2026.1747411 (PMC12953097; doi:10.3389/fimmu.2026.1747411)
Supplement: Supplementary file 5 [file Table1.pdf]

Supplementary Table 1. Sensitivity analysis of subgroups in this study.

CMV	Ashimine et al. (2014)	0.97721242(0.81331774-1.1741341)
	Fuchinoue et al. (2011)	1.0086915(0.83818854-1.2138779)
	Hamano et al. (2020)	1.0098007(0.84205436-1.210964)
	Hatakeyama et al. (2014)	1.012877(0.84598074-1.2126987)
	Hwang et al. (2013)	1.0203712(0.84970057-1.2253226)
	Ko et al. (2020)	1.0633681(0.81983616-1.3792412)
	Kohei et al. (2011)	1.0227931(0.85410147-1.2248027)
	Okumi et al. second area (2016)	0.95479852(0.787275551-0.1579684)
	Park et al. (2016)	1.0020445(0.83740428-1.1990543)
	Park et al. (2016)	1.0020445(0.83740428-1.1990543)
	Prabhakar et al. (2021)	1.0143297(0.84707007-1.2146158)
	Shin et al. (2015)	1.007012(0.8410434-1.2057322)
	Song et al. (2017)	0.95397192(0.79380852-1.1464508)
	Combined	1.0020445(0.83740428-1.1990543)
BK	Hatakeyama et al. (2014)	1.1355576(0.89524965-1.4403703)
	Hamano et al. (2020)	1.1239097(0.88264194-1.4311272)
	Hwang et al. (2013)	1.0595099(0.83162421-1.349842)
	Ko et al. (2020)	1.1942298(0.72562878-1.9654467)
	Prabhakar et al. (2021)	1.1267739(0.88489895-1.434762)
	Shin et al. (2015)	1.105664(0.86947769-1.4060084)
	Song et al. (2017)	1.162816(0.91077254-1.484609)
	Combined	1.1211744(0.88487212-1.4205804)
Acute Rejection	Ashimine et al. (2014)	1.224508(0.93580543-1.6022772)
	Fuchinoue et al. (2011)	1.2770511(1.0062016-1.6208078)
	Hamano et al. (2020)	1.1891125(0.9087481-1.555974)
	Hatakeyama et al. (2014)	1.188702(0.91088063-1.5512597)
	Hwang et al. (2013)	1.1673377(0.88451854-1.5405865)
	Jeon et al. (2010)	1.2480752(0.96994357-1.6059613)
	Jiang et al. (2015)	1.1938414(0.88802058-1.6049823)
	Kim et al. (2020)	1.1486575(0.87240571-1.5123858)
	Ko et al. (2017)	1.1923951(0.87558074-1.6238436)
	Ko et al. (2020)	1.1737202(0.86507777-1.5924802)
	Kohei et al. (2011)	1.2587616(0.97789751-1.6202933)
	Okumi et al. second area (2016)	1.2056936(0.917280761-0.5847896)
	Park et al. (2016)	1.1932227(0.91294259-1.5595509)
	Prabhakar et al. (2021)	1.1577919(0.87141409-1.5382836)
	Shin et al. (2015)	1.2677654(0.96599759-1.6638023)
	Song et al. (2017)	1.1595837(0.87054396-1.544591)
	Tanabe et al. (2009)	1.2558637(0.9688891-1.6278372)
	Wang et al. (2020)	1.1856191(0.8914094-1.5769326)
	Combined	1.2065059(0.92482783-1.5739756)
Urinary Tract Infection	Kim et al. (2020)	0.66094859(0.43054967-1.0146403)
	Ko et al. (2020)	0.76561044(0.38994858-1.5031709)
	Prabhakar et al. (2021)	0.78953294(0.42373215-1.4711234)
	Shin et al. (2015)	0.84338936(0.58713781-1.2114798)
	Song et al. (2017)	0.69430739(0.40775588-1.1822337)
	Combined	0.75907571(0.48267474-1.1937561)
Graft Survival Rate		
1-year	Ashimine et al. (2014)	0.51822156(0.38631168-0.69517336)
	Fuchinoue et al. (2011)	0.51509832(0.38340431-0.69202739)
	Hatakeyama et al. (2014)	0.51822156(0.38631168-0.69517336)
	Hwang et al. (2013)	0.51079533(0.37970866-0.68713701)
	Jeon et al. (2010)	0.51822156(0.38631168-0.69517336)
	Jiang et al. (2020)	0.51337305(0.38187308-0.6901557)
	Ko et al. (2020)	0.51895309(0.38512848-0.69927915)

	Kohei et al. (2011)	0.52321484(0.38934971-0.7031051)
	Park et al. (2016)	0.51822156(0.38631168-0.69517336)
	Park et al. (2016)	0.51822156(0.38631168-0.69517336)
	Shin et al. (2015)	0.52369008(0.38963263-0.70387149)
	Takahashi et al. (2013)	0.59039513(0.25698209-1.3563841)
	Tanabe et al. (2009)	0.51943718(0.38657303-0.69796639)
	Wang et al. (2020)	0.51216529(0.38100527-0.68847679)
	Yu et al. (2017)	0.51822156(0.38631168-0.69517336)
	Combined	0.51822156(0.38631168-0.69517336)
3-year	Ashimine et al. (2014)	1.307086(0.76257722-2.2403946)
	Fuchinoue et al. (2011)	1.1294264(0.64767772-1.969504)
	Hatakeyama et al. (2014)	1.3040448(0.75515907-2.2518869)
	Jeon et al. (2010)	1.307086(0.76257722-2.2403946)
	Jiang et al. (2020)	1.5089126(0.84240148-2.7027696)
	Ko et al. (2017)	1.3952267(0.76688091-2.5384091)
	Ko et al. (2020)	1.4828527(0.81864897-2.6859523)
	Shin et al. (2015)	1.3347907(0.76527338-2.3281434)
	Song et al. (2017)	1.3458768(0.75487747-2.399574)
	Wang et al. (2020)	1.2106456(0.69299871-2.1149575)
	Yu et al. (2017)	1.1113337(0.61900547-1.9952371)
	Combined	1.307086(0.76257722-2.2403946)
5-year	Fuchinoue et al. (2011)	0.48276968(0.26130892-0.89191967)
	Hamano et al. (2020)	0.5243236(0.26807163-1.025529)
	Hatakeyama et al. (2014)	0.60580949(0.29106456-1.2609063)
	Jiang et al. (2020)	0.75382086(0.33107163-1.7163835)
	Ko et al. (2020)	0.62258755(0.2918576-1.3280972)
	Kohei et al. (2011)	0.47284852(0.25160686-0.88863127)
	Okumi et al.second area (2016)	0.67211755(0.2914592-1.5499322)
	Prabhakar et al. (2021)	0.80214133(0.3237304-1.9875511)
	Setoguchi et al. (2007)	0.67787884(0.32305203-1.4224326)
	Takahashi et al. (2013)	0.84533605(0.34166314-2.0915134)
	Combined	0.62272987(0.30543648-1.2696338)
Patient Survival Rate		
1-year	Ashimine et al. (2014)	0.96854737(0.67905324-1.3814587)
	Fuchinoue et al. (2014)	0.96854737(0.67905324-1.3814587)
	Hatakeyama et al. (2013)	0.96854737(0.67905324-1.3814587)
	Hwang et al. (2010)	1.0077822(0.70091465-1.4489996)
	Jeon et al. (2017)	0.96854737(0.67905324-1.3814587)
	Jiang et al. (2020)	0.96854737(0.67905324-1.3814587)
	Ko et al. (2020)	0.96668841(0.67124787-1.392163)
	Kohei et al. (2011)	0.96854737(0.67905324-1.3814587)
	Okumi et al. second area (2016)	0.93475839(0.653218481-0.3376432)
	Park et al. (2016)	0.96854737(0.67905324-1.3814587)
	Shin et al. (2015)	1.003509(0.697403-1.4439718)
	Takahashi et al. (2013)	0.72285593(0.35149836-1.4865523)
	Tanabe et al. (2009)	0.96854737(0.67905324-1.3814587)
	Wang et al. (2020)	0.98985857(0.68914936-1.4217818)
	Yu et al. (2017)	0.97799245(0.67949219-1.4076236)
	Combined	0.96854737(0.67905324-1.3814587)
3-year	Ashimine et al. (2014)	0.59197321(0.35225696-0.99482004)
	Fuchinoue et al. (2011)	0.55153396(0.33282261-0.91396949)
	Hatakeyama et al. (2014)	0.55153396(0.33282261-0.91396949)
	Jeon et al. (2010)	0.55153396(0.33282261-0.91396949)
	Jiang et al. (2020)	0.5558633(0.33247952-0.92933244)
	Ko et al. (2017)	0.65668931(0.36613415-1.177822)

	Ko et al. (2020)	0.49688945(0.29274973-0.84337951)
	Kohei et al. (2011)	0.55153396(0.33282261-0.91396949)
	Okumi et al. second area (2016)	0.471065(0.27503231-0.80682242)
	Shin et al. (2015)	0.58272687(0.33933611-1.000691)
	Song et al. (2017)	0.55521032(0.32812354-0.93945866)
	Tanabe et al. (2009)	0.55153396(0.33282261-0.91396949)
	Wang et al. (2020)	0.53672131(0.31748257-0.90735616)
	Yu et al. (2017)	0.54348622(0.31111252-0.94942262)
	Combined	0.55153396(0.33282261-0.91396949)
5-year	Fuchinoue et al. (2014)	1.1985879(0.9158657-1.5685846)
	Hamano et al. (2020)	1.2177812(0.92616672-1.601214)
	Hatakeyama et al. (2011)	1.1985879(0.9158657-1.5685846)
	Jiang et al. (2020)	1.2221541(0.93021137-1.6057216)
	Ko et al. (2021)	1.2146651(0.92620657-1.5929614)
	Kohei et al. (2016)	1.1985879(0.9158657-1.5685846)
	Okumi et al. second area (2007)	1.1896448(0.905471361-0.5630034)
	Setoguchi et al. (2013)	1.205487(0.91948685-1.5804457)
	Takahashi et al. (2020)	0.76532258(0.34450124-1.7001932)
	Combined	1.1985879(0.9158657-1.5685846)
