# Supplementary material for: The viability of ABO-incompatible kidney transplants: a single-center cohort in China
Source: Front Immunol. 2026 Feb 17;17:1747411. doi: 10.3389/fimmu.2026.1747411 (PMC12953097; doi:10.3389/fimmu.2026.1747411)
Supplement: Supplementary file 6 [file Table2.pdf]

Supplementary Table 2. The egger's test of researches of this meta-analysis.

	Egger's test P > |t|
1-year graft survival rate	0.727
3-year graft survival rate	0.334
5-year graft survival rate	0.014
1-year patient survival rate	0.307
3-year patient survival rate	0.907
5-year patient survival rate	0.071
Cytomegalovirus	0.535
BK Virus	0.746
Urinary Tract Infection	0.905
Acute Rejection	0.349
