# Supplementary material for: The viability of ABO-incompatible kidney transplants: a single-center cohort in China
Source: Front Immunol. 2026 Feb 17;17:1747411. doi: 10.3389/fimmu.2026.1747411 (PMC12953097; doi:10.3389/fimmu.2026.1747411)
Supplement: Supplementary file 7 [file Table3.pdf]

Supplementary Table 3. Analysis of postoperative renal function in ABOi-LDKT and ABOc-LDKT groups.

Postoperative duration	Renal Function	ABOi-LDKT (N=41)	ABOc-LDKT (N=132)	P
1st week	Scr	135.20 ± 59.50	149.74 ± 99.13	0.264
	GFR	66.82 ± 25.35	64.89 ± 26.88	0.683
2nd week	Scr	130.90 ± 49.37	171.67 ± 111.78	0.002*
	GFR	68.21 ± 26.04	58.99 ± 27.70	0.061
1st month	Scr	145.32 ± 62.24	162.18 ± 82.57	0.174
	GFR	61.49 ± 23.79	56.11 ± 21.37	0.210
6th month	Scr	135.20 ± 39.68	148.15 ± 52.82	0.114
	GFR	62.47 ± 21.36	58.80 ± 19.06	0.356
1st year	Scr	136.24 ± 33.65	137.67 ± 51.20	0.845
	GFR	59.22 ± 16.12	64.78 ± 23.92	0.112
3rd year	Scr	136.12 ± 45.62	135.52 ± 43.02	0.953
	GFR	61.41 ± 21.58	63.39 ± 22.35	0.689
